# Is SIRT6 Activity Neuroprotective and How Does It Differ from SIRT1 in This Regard?

**DOI:** 10.3389/fncel.2017.00165

**Published:** 2017-06-08

**Authors:** Bor L. Tang

**Affiliations:** ^1^Department of Biochemistry, Yong Loo Lin School of Medicine, National University Health SystemSingapore, Singapore; ^2^NUS Graduate School for Integrative Sciences and Engineering, National University of SingaporeSingapore, Singapore

**Keywords:** SIRT6, SIRT1, neuroprotection, Alzheimer's disease, oxidative stress

## Introduction

The NAD^+^-dependent type III histone deacetylases, or Sirtuins, have differing cellular localizations, and can have nuclear (SIRT1 and SIRT6), cytoplasmic (SIRT2), mitochondrial (SIRT3, SIRT4, and SIRT5) or nucleolar (SIRT7) localization. The founding member of the Sirtuin family, *S. cerevisiae* Sir2p, and its orthologs have important roles in senescence and aging, with manipulation of their activities resulting in modest lifespan extension, as verified in several model organisms. Sirtuins have a wide range of nuclear and cytoplasmic substrates, and are implicated in the transcriptional and epigenetic regulations of cellular and systemic processes of energy metabolism, stress response, death and survival, as well as pathological conditions such as malignancy (Chalkiadaki and Guarente, 2015) and neurodegeneration (Herskovits and Guarente, 2013). Perhaps the most extensively studied member of the mammalian Sirtuin paralogs is SIRT1, but the cellular and systemic roles of other paralogs have also been intensively investigated in recent years.

SIRT6, like SIRT1, is nuclear localized. Acting primarily as a histone deacetylase, earlier studies have delineated its roles in telomere maintenance (Michishita et al., 2008), DNA repair (Mao et al., 2011) and transcription regulation of glucose homeostasis (Zhong et al., 2010). The importance of SIRT6 in mammals is illustrated by the fact that SIRT6 deficient mice have retarded postnatal development associated with cellular genomic instability, systemic metabolic defects, and exhibited premature senescence and aging, with early death occurring at around 4 weeks after birth (Mostoslavsky et al., 2006). In the past several years, many other emerging physiological as well as pathological functions for SIRT6 in multiple tissue contexts have also been reported (Tasselli et al., 2017). These include the regulation of the circadian clock (Masri et al., 2014), tumor suppression (Kugel et al., 2016) as well as lifespan extension in mammals (Kanfi et al., 2012), among others. In many of these cases, SIRT6 functions at first glance appear to have significant overlaps with that of SIRT1.

Unlike SIRT1, which has been extensively studied with regards to its roles in neurons and the central nervous system (CNS) (Herskovits and Guarente, 2014; Ng et al., 2015), the function of SIRT6 in neural tissues has been less well explored. Like SIRT1, SIRT6 appears to have anti-aging activities through the regulation of inflammation (Kawahara et al., 2009), the expression of antioxidant genes (Pan et al., 2016) as well as proteasomal degradation of senescence factors (Zhao et al., 2016), all of which are related to neuroprotection. Like SIRT1, SIRT6 activity may therefore be largely neuroprotective, and this expectation appears to have been borne out by several recent reports, which suggest that loss of SIRT6 during aging and neuropathological settings contributes to neurodegenerative processes. However, elevated SIRT6 level has also been noted to be undesirable under certain conditions. SIRT6 may also differ from SIRT1 in terms of enzymatic activity and mechanism of action. In the paragraphs below, I shall explore current evidence (as well as counterevidence) for the neuroprotective potential of SIRT6, and draw comparisons with that of SIRT1.

## Evidence for SIRT6's role in neuroprotection

Like SIRT1, SIRT6 is abundantly expressed in mammalian brain neurons (Lee et al., 2013; Cardinale et al., 2015). However, while SIRT1 levels tend to increase in the aged brain (Koltai et al., 2010; Braidy et al., 2015), the levels of SIRT6 decline significantly with age (Braidy et al., 2015). Several recent reports have also implicated a neuroprotective function for SIRT6 in diverse neuropathological settings. SIRT6 is highly expressed in the retina, and analysis of SIRT6 knockout (KO) mice revealed retinal neural transmission defects that are associated with a down-regulation of the levels of both ionotropic and metabotropic glutamate receptors, and consequently impaired retinal function (Silberman et al., 2014). Investigating an Alzheimer's disease (AD) mouse model [transgenic for five familial AD (5XFAD) mutations associated with the Amyloid precursor protein (APP) and Presenilin 1], as well as brain samples from AD patients, Jung and colleagues reported a reduction in SIRT6 protein in these models (Jung et al., 2016). The amyloid plaque forming Aβ42 peptide downregulated SIRT6 levels in mouse cortical neurons as well as the mouse hippocampal cell line HT22, and this downregulation corresponded with an increase in the acetylation of histone K9 and K56. Interestingly, Aβ42, at least in HT22 cells, appeared to have suppressed SIRT6 through a c-Jun N-terminal kinase (JNK) and p53-dependent mechanism. In line with SIRT6's known role in facilitating DNA repair, another of the authors' findings is that SIRT6 over-expression attenuated Aβ42-induced DNA damage, as assessed by the H2AX phosphorylation, a marker for DNA double strand breaks (Jung et al., 2016).

Another recent study investigated the role of SIRT6 using mice with conditional knockout of SIRT6 in the brain (brS6KO mice, with Nestin-Cre based deletion of exon2-3 floxed SIRT6). These mice were previously shown to have stunted postnatal growth, but ultimately become obese with time (Schwer et al., 2010). Kaluski et al. further showed that the brS6KO mouse brain exhibited increased levels of the DNA repair protein Ataxia telangiectasia mutated (ATM), H2AX phosphorylation, TUNEL-labeled cells in the cortex, as well as impaired contextual learning that is suggestive of neurodegeneration (Kaluski et al., 2017). Indeed, the authors found that loss of SIRT6 led to tau hyperphosphorylation, which correlated with a decreased in glucose synthase kinase 3 (GSK3) phosphorylation (therefore increased GSK activity), with the latter restored with SIRT6 re-expression. Like Jung et al. above, the authors also found that SIRT6 levels are markedly reduced in human AD brain samples (Kaluski et al., 2017). Taken together, these studies suggest that SIRT6 reduction during diseased conditions (such as AD) may further enhance neuronal death and degeneration.

With regards to more acute neuronal injuries, evidence for a direct protective effect of SIRT6 on CNS neurons is lacking. On the other hand, a more definitive role for SIRT1 in protection against brain ischemic injury has been demonstrated, with a significantly increased in infarct volume in *SIRT1*^−/−^ mice compared to control (Hernández-Jiménez et al., 2013). Several findings nonetheless point toward the notion that SIRT6 activity may well be congruent with, if not analogous to, SIRT1 in ischemic injury. In models of middle cerebral artery occlusion and oxygen-glucose deprivation, SIRT6 reduction may promote the release of the proinflammatory high mobility group box-1 (HMGB1) protein (Lee et al., 2013), possibly resulting in heightening of inflammation. SIRT6 was also shown to mediate the protective effect of the gasotransmitter hydrogen sulfide's positive effects on oxygen-glucose deprivation and reperfusion-stimulated brain endothelial cells (Hu et al., 2015).

The above findings suggest that maintaining or increasing SIRT6 activities, particularly in the aging brain, may be therapeutically beneficial against neurodegenerative disorders and CNS injury.

## SIRT6's adverse effects in the neuronal context

Although the notion that SIRT6 is neuroprotective may seem somewhat intuitive, there exist some experimental findings that are against it. In assessing the effect of Sirtuin paralog over-expressions in rescuing cerebellar granule neurons (CGNs) from low potassium treatment, it was found that in contrast to SIRT1, SIRT6 over-expression reduced the viability of CGNs (Pfister et al., 2008). Another investigation revealed that SIRT6 over-expression in cultured mouse cortical neurons stressed with the oxidant H_2_O_2_ further reduced neuronal survival (Cardinale et al., 2015). In these types of neurons, this finding with SIRT6 was in agreement with earlier findings associated with SIRT1, as both SIRT1 inhibition and its silencing was also shown to result in worsened viability during H_2_O_2_ treatment (Li et al., 2008). With human neuroblastoma SH-SY5Y cells, SIRT6 over-expression was also shown to decrease cell viability under H_2_O_2_-induced oxidative stress, which was associated with increased ROS production and autophagy induction (Shao et al., 2016).

In yet another report, SIRT6 over-expression in rat hippocampal neurons was found to attenuate AKT-GSK-3β phosphorylation (Mao et al., 2011), both of which are components of an important survival signaling pathway in ischemic (Endo et al., 2006) and neurotoxic (Mariottini et al., 2009) brain injury. This attenuation is associated with the animals acquiring depression-like behaviors (Mao et al., 2017). Over-expression of SIRT6 in the hippocampus CA1 region of rats impaired the formation of long-term contextual fear memory (but not short-term fear memory) (Yin et al., 2016). Other than the potential in impairing neuronal survival under certain pathological situations, excessive SIRT6 activity may therefore also be undesirable in some neurological contexts. One should of course be careful in interpreting results from over-expression experiments, as these are prone to non-specific, gain of function effects.

## Context-dependent beneficial effects of SIRT6 expression and activity in the CNS—a comparison with SIRT1

From the discussion above it appears that the neuroprotective effect of SIRT6 may be somewhat context-dependent rather than universal. The basis of this context dependency is not yet clear. However, it is notable that context-dependence of neuroprotection was also observed to some degree with SIRT1, and it would thus be of interest to see if these occur via similar mechanisms. SIRT1's role in AD is well known (Wong and Tang, 2016). Like SIRT6, SIRT1 activity decreases tau phosphorylation, and this occurs via its direct interaction with and deacetylation of tau, which reduced the latter's phosphorylation and promoted ubiquitination and turnover (Min et al., 2010). Whether SIRT6 has a similar activity on tau remains to be seen. SIRT activity has also been shown to promote non-amyloidogenic α-secretase mediated APP cleavage, likely acting via a major substrate Peroxisome proliferator-activated receptor-α (PPARα) (Lee et al., 2014; Corbett et al., 2015). Again, whether SIRT6 could promote non-amyloidogenic APP cleavage remains to be investigated.

It has been previously argued that while sustained SIRT1 activity may benefit chronic stresses associated with neurodegenerative disorders, elevation of SIRT1 activity or levels may instead adversely affect acutely injured neurons (Ng and Tang, 2013). One mechanism by which this could occur is an antagonistic competition between SIRT1 and the DNA repair enzyme Poly (ADP-ribose) polymerase 1 (PARP-1) for the same cofactor NAD^+^, which may become limiting in stressed conditions (Liu et al., 2009). In this regard, SIRT6 may act differently from SIRT1 as it has an ADP-ribosyl transferase activity, while the latter is primarily a deacetylase. During oxidative stress, SIRT6 has been shown to promote DNA repair by activating PARP-1 via ADP-ribosylation of the latter on Lys 521 (Mao et al., 2011). In this regard, SIRT6 could be more of a promoter of PARP-1 activity during injury than SIRT1. Working out the molecular interplay between these two nuclear Sirtuins and PARP-1 under various conditions of stress would be important.

Other than intrinsic enzymatic activity and differential substrate preference, there are also other important differences between CNS SIRT6 and SIRT1, for example the spatial and temporal difference in their expression, as well as substrate specificity. As mentioned earlier, SIRT6 levels decline in the aging brain as oppose to SIRT1's increase. SIRT6 levels are in fact positively regulated by SIRT1 and FOXO3a in metabolically active peripheral tissues (Kim et al., 2010). Interestingly, while SIRT1 levels in muscles were elevated by physical training, the levels of SIRT6 were instead attenuated (Koltai et al., 2010). SIRT1 and SIRT6 have overlapping activities and localizations at the cellular level and in the CNS tissues. Common activators and inhibitors would likely affect both to some degree, and this complicates attempts to understand their respective roles in various neurological settings and their engagement of downstream substrates. A better understanding the differences and crosstalk between these two nuclear Sirtuins would therefore be important for their exploitations as specific therapeutic targets for neurodegenerative disorders and brain injury.

## Author contributions

The author confirms being the sole contributor of this work and approved it for publication.

### Conflict of interest statement

The author declares that the research was conducted in the absence of any commercial or financial relationships that could be construed as a potential conflict of interest.

## References

[B1] Braidy N. Poljak A. Grant R. Jayasena T. Mansour H. Chan-Ling T.. (2015). Differential expression of sirtuins in the aging rat brain. Front. Cell. Neurosci. 9:167. 10.3389/fncel.2015.00167 26005404 PMC4424846

[B2] Cardinale A. de Stefano M. C. Mollinari C. Racaniello M. Garaci E. Merlo D. (2015). Biochemical characterization of sirtuin 6 in the brain and its involvement in oxidative stress response. Neurochem. Res. 40, 59–69. 10.1007/s11064-014-1465-1 25366464

[B3] Chalkiadaki A. Guarente L. (2015). The multifaceted functions of sirtuins in cancer. Nat. Rev. Cancer 15, 608–624. 10.1038/nrc3985 26383140

[B4] Corbett G. T. Gonzalez F. J. Pahan K. (2015). Activation of peroxisome proliferator-activated receptor α stimulates ADAM10-mediated proteolysis of APP. Proc. Natl. Acad. Sci. U.S.A. 112, 8445–8450. 10.1073/pnas.1504890112 26080426 PMC4500265

[B5] Endo H. Nito C. Kamada H. Nishi T. Chan P. H. (2006). Activation of the Akt/GSK3beta signaling pathway mediates survival of vulnerable hippocampal neurons after transient global cerebral ischemia in rats. J. Cereb. Blood Flow Metab. 26, 1479–1489. 10.1038/sj.jcbfm.9600303 16538228

[B6] Hernández-Jiménez M. Hurtado O. Cuartero M. I. Ballesteros I. Moraga A. Pradillo J. M.. (2013). Silent information regulator 1 protects the brain against cerebral ischemic damage. Stroke 44, 2333–2337. 10.1161/STROKEAHA.113.001715 23723308

[B7] Herskovits A. Z. Guarente L. (2013). Sirtuin deacetylases in neurodegenerative diseases of aging. Cell Res. 23, 746–758. 10.1038/cr.2013.70 23689277 PMC3674397

[B8] Herskovits A. Z. Guarente L. (2014). SIRT1 in neurodevelopment and brain senescence. Neuron 81, 471–483. 10.1016/j.neuron.2014.01.028 24507186 PMC4040287

[B9] Hu Y. Li R. Yang H. Luo H. Chen Z. (2015). Sirtuin 6 is essential for sodium sulfide-mediated cytoprotective effect in ischemia/reperfusion-stimulated brain endothelial cells. J. Stroke Cerebrovasc. Dis. 24, 601–609. 10.1016/j.jstrokecerebrovasdis.2014.10.006 25543188

[B10] Jung E. S. Choi H. Song H. Hwang Y. J. Kim A. Ryu H.. (2016). p53-dependent SIRT6 expression protects Aβ42-induced DNA damage. Sci. Rep. 6:25628. 10.1038/srep25628 27156849 PMC4860716

[B11] Kaluski S. Portillo M. Besnard A. Stein D. Einav M. Zhong L.. (2017). Neuroprotective functions for the histone deacetylase SIRT6. Cell Rep. 18, 3052–3062. 10.1016/j.celrep.2017.03.008 28355558 PMC5389893

[B12] Kanfi Y. Naiman S. Amir G. Peshti V. Zinman G. Nahum L.. (2012). The sirtuin SIRT6 regulates lifespan in male mice. Nature 483, 218–221. 10.1038/nature10815 22367546

[B13] Kawahara T. L. A. Michishita E. Adler A. S. Damian M. Berber E. Lin M.. (2009). SIRT6 links histone H3 lysine 9 deacetylation to NF-kappaB-dependent gene expression and organismal life span. Cell 136, 62–74. 10.1016/j.cell.2008.10.052 19135889 PMC2757125

[B14] Kim H. S. Xiao C. Wang R. H. Lahusen T. Xu X. Vassilopoulos A.. (2010). Hepatic-specific disruption of SIRT6 in mice results in fatty liver formation due to enhanced glycolysis and triglyceride synthesis. Cell Metab. 12, 224–236. 10.1016/j.cmet.2010.06.009 20816089 PMC2935915

[B15] Koltai E. Szabo Z. Atalay M. Boldogh I. Naito H. Goto S.. (2010). Exercise alters SIRT1, SIRT6, NAD and NAMPT levels in skeletal muscle of aged rats. Mech. Ageing Dev. 131, 21–28. 10.1016/j.mad.2009.11.002 19913571 PMC2872991

[B16] Kugel S. Sebastián C. Fitamant J. Ross K. N. Saha S. K. Jain E.. (2016). SIRT6 suppresses pancreatic cancer through control of lin28b. Cell 165, 1401–1415. 10.1016/j.cell.2016.04.033 27180906 PMC4892983

[B17] Lee H. R. Shin H. K. Park S. Y. Kim H. Y. Lee W. S. Rhim B. Y.. (2014). Cilostazol suppresses β-amyloid production by activating a disintegrin and metalloproteinase 10 via the upregulation of SIRT1-coupled retinoic acid receptor-β. J. Neurosci. Res. 92, 1581–1590. 10.1002/jnr.23421 24903973

[B18] Lee O. H. Kim J. Kim J. M. Lee H. Kim E. H. Bae S. K.. (2013). Decreased expression of sirtuin 6 is associated with release of high mobility group box-1 after cerebral ischemia. Biochem. Biophys. Res. Commun. 438, 388–394. 10.1016/j.bbrc.2013.07.085 23899523

[B19] Li Y. Xu W. McBurney M. W. Longo V. D. (2008). SirT1 inhibition reduces IGF-I/IRS-2/Ras/ERK1/2 signaling and protects neurons. Cell Metab. 8, 38–48. 10.1016/j.cmet.2008.05.004 18590691 PMC2822839

[B20] Liu D. Gharavi R. Pitta M. Gleichmann M. Mattson M. P. (2009). Nicotinamide prevents NAD+ depletion and protects neurons against excitotoxicity and cerebral ischemia: NAD+ consumption by SIRT1 may endanger energetically compromised neurons. Neuromolecular Med. 11, 28–42. 10.1007/s12017-009-8058-1 19288225 PMC2677622

[B21] Mao Q. Gong X. Zhou C. Tu Z. Zhao L. Wang L.. (2017). Up-regulation of SIRT6 in the hippocampus induced rats with depression-like behavior via the block Akt/GSK3β signaling pathway. Behav. Brain Res. 323, 38–46. 10.1016/j.bbr.2017.01.035 28130175

[B22] Mao Z. Hine C. Tian X. Van Meter M. Au M. Vaidya A.. (2011). SIRT6 promotes DNA repair under stress by activating PARP1. Science 332, 1443–1446. 10.1126/science.1202723 21680843 PMC5472447

[B23] Mariottini C. Scartabelli T. Bongers G. Arrigucci S. Nosi D. Leurs R.. (2009). Activation of the histaminergic H3 receptor induces phosphorylation of the Akt/GSK-3 beta pathway in cultured cortical neurons and protects against neurotoxic insults. J. Neurochem. 110, 1469–1478. 10.1111/j.1471-4159.2009.06249.x 19549072

[B24] Masri S. Rigor P. Cervantes M. Ceglia N. Sebastian C. Xiao C.. (2014). Partitioning circadian transcription by SIRT6 leads to segregated control of cellular metabolism. Cell 158, 659–672. 10.1016/j.cell.2014.06.050 25083875 PMC5472354

[B25] Michishita E. McCord R. A. Berber E. Kioi M. Padilla-Nash H. Damian M.. (2008). SIRT6 is a histone H3 lysine 9 deacetylase that modulates telomeric chromatin. Nature 452, 492–496. 10.1038/nature06736 18337721 PMC2646112

[B26] Min S. W. Cho S. H. Zhou Y. Schroeder S. Haroutunian V. Seeley W. W.. (2010). Acetylation of tau inhibits its degradation and contributes to tauopathy. Neuron 67, 953–966. 10.1016/j.neuron.2010.08.044 20869593 PMC3035103

[B27] Mostoslavsky R. Chua K. F. Lombard D. B. Pang W. W. Fischer M. R. Gellon L.. (2006). Genomic instability and aging-like phenotype in the absence of mammalian SIRT6. Cell 124, 315–329. 10.1016/j.cell.2005.11.044 16439206

[B28] Ng F. Tang B. L. (2013). When is Sirt1 activity bad for dying neurons? Front. Cell. Neurosci. 7:186. 10.3389/fncel.2013.00186 24167473 PMC3807049

[B29] Ng F. Wijaya L. Tang B. L. (2015). SIRT1 in the brain-connections with aging-associated disorders and lifespan. Front. Cell. Neurosci. 9:64. 10.3389/fncel.2015.00064 25805970 PMC4353374

[B30] Pan H. Guan D. Liu X. Li J. Wang L. Wu J.. (2016). SIRT6 safeguards human mesenchymal stem cells from oxidative stress by coactivating NRF2. Cell Res. 26, 190–205. 10.1038/cr.2016.4 26768768 PMC4746611

[B31] Pfister J. A. Ma C. Morrison B. E. D'Mello S. R. (2008). Opposing effects of sirtuins on neuronal survival: SIRT1-mediated neuroprotection is independent of its deacetylase activity. PLoS ONE 3:e4090. 10.1371/journal.pone.0004090 19116652 PMC2605257

[B32] Schwer B. Schumacher B. Lombard D. B. Xiao C. Kurtev M. V. Gao J.. (2010). Neural sirtuin 6 (Sirt6) ablation attenuates somatic growth and causes obesity. Proc. Natl. Acad. Sci. U.S.A. 107, 21790–21794. 10.1073/pnas.1016306107 21098266 PMC3003110

[B33] Shao J. Yang X. Liu T. Zhang T. Xie Q. R. Xia W. (2016). Autophagy induction by SIRT6 is involved in oxidative stress-induced neuronal damage. Protein Cell 7, 281–290. 10.1007/s13238-016-0257-6 26983852 PMC4818841

[B34] Silberman D. M. Ross K. Sande P. H. Kubota S. Ramaswamy S. Apte R. S.. (2014). SIRT6 is required for normal retinal function. PLoS ONE 9:e98831. 10.1371/journal.pone.0098831 24896097 PMC4045872

[B35] Tasselli L. Zheng W. Chua K. F. (2017). SIRT6: novel mechanisms and links to aging and disease. Trends Endocrinol. Metab. 28, 168–185. 10.1016/j.tem.2016.10.002 27836583 PMC5326594

[B36] Wong S. Y. Tang B. L. (2016). SIRT1 as a therapeutic target for Alzheimer's disease. Rev. Neurosci. 27, 813–825. 10.1515/revneuro-2016-0023 27497424

[B37] Yin X. Gao Y. Shi H. S. Song L. Wang J. C. Shao J.. (2016). Overexpression of SIRT6 in the hippocampal CA1 impairs the formation of long-term contextual fear memory. Sci. Rep. 6:18982. 10.1038/srep18982 26732053 PMC4702175

[B38] Zhao G. Wang H. Xu C. Wang P. Chen J. Wang P.. (2016). SIRT6 delays cellular senescence by promoting p27Kip1 ubiquitin-proteasome degradation. Aging 8, 2308–2323. 10.18632/aging.101038 27794562 PMC5115890

[B39] Zhong L. D'Urso A. Toiber D. Sebastian C. Henry R. E. Vadysirisack D. D.. (2010). The histone deacetylase Sirt6 regulates glucose homeostasis via Hif1alpha. Cell 140, 280–293. 10.1016/j.cell.2009.12.041 20141841 PMC2821045

